# *Cymodocea nodosa*, a Promising Seagrass of Nutraceutical Interest: Overview of Phytochemical Constituents and Potential Therapeutic Uses

**DOI:** 10.3390/nu17071236

**Published:** 2025-04-01

**Authors:** Marinella De Leo, Lidia Ciccone, Virginia Menicagli, Elena Balestri, Alessandra Braca, Paola Nieri, Lara Testai

**Affiliations:** 1 Department of Pharmacy, University of Pisa, 56126 Pisa, Italy; marinella.deleo@unipi.it (M.D.L.); lidia.ciccone@unipi.it (L.C.); alessandra.braca@unipi.it (A.B.); paola.nieri@unipi.it (P.N.); 2 Marine Pharmacology Interdepartmental Center, University of Pisa, 56126 Pisa, Italy; elena.balestri@unipi.it; 3 Interdepartmental Center Nutrafood “Nutraceuticals and Food for Health”, Interdepartmental Center, University of Pisa, 56124 Pisa, Italy; 4 Center for Instrument Sharing of the University of Pisa (CISUP), University of Pisa, 56126 Pisa, Italy; 5 Department of Biology, University of Pisa, 56126 Pisa, Italy; virginia.menicagli@biologia.unipi.it

**Keywords:** *Cymodocea nodosa*, seagrass, bioactive compounds, marine nutraceuticals, specialized metabolites

## Abstract

Background/Objectives: Seagrasses are marine angiosperms capable of completing their life cycle in water; they have been used as food source and biomass for producing fertilizer, but their potential nutritional and health-promoting properties have been largely overlooked. *Cymodocea nodosa* (Ucria) Ascherson (family Cymodoceaceae) is emerging as one of the most interesting seagrass species due to its content in health promoting substances. Methods: In this review article, a revision of the literature on phytochemical constituents and the main potential therapeutic uses of *C. nodosa* was carried out. Results: Despite the growing interest in *C. nodosa* for its key ecological role and for being a potential source of bioactive compounds, comprehensive chemical studies about its composition are still limited. Compounds reported as *C. nodosa* constituents include fatty acids, phytosterols, polysaccharides, phenolic acids, hydroxycinnamic acids, flavonoid glycosides, terpenoids, and diarylheptanoids. As concerns potential therapeutic uses, *C. nodosa* extract, both polyphenolic and polysaccharidic, might be useful for the management of metabolic disorders, which is currently the most documented in addition to the antioxidant action. Conclusions: *Cymodocea nodosa* emerges as one of the most promising seagrass species as a source of bioactive compounds and for its potential in maintaining health status.

## 1. Introduction

Seagrasses are aquatic angiosperms adapted to live in the marine environment and are currently distributed in coastal areas of all continents from 0 to approximately 50 m depth [1]. These plants are considered as ecosystem engineers due to their ability to form extensive and highly productive meadows [2], which play countless and valuable ecological functions in coastal environments, from supporting biodiversity by providing food, shelter, and nursery habitats to improving water quality and protecting coastline [3,4,5].

**Figure 1:**
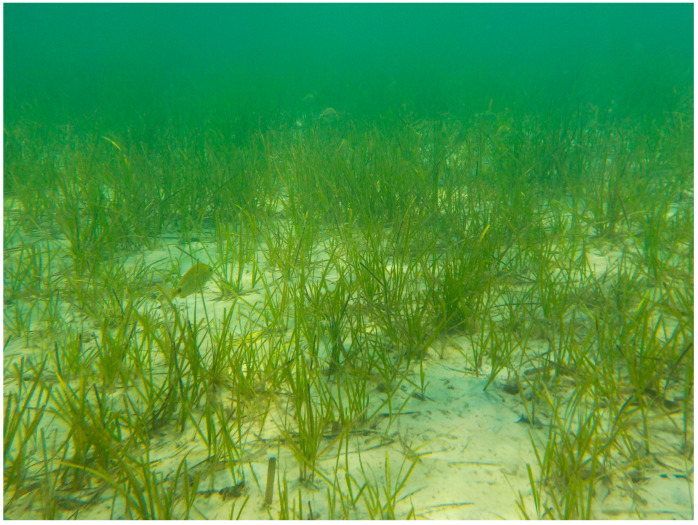
The photo represents specimens of *Cymodocea nodosa* of the Liguria sea (Italy).

Seagrasses have been exploited by humans for centuries for many purposes. For example, their leaves have been employed in traditional medicine, and as soil amendment or fertilizers, cattle feed (in particular for marine organisms) and filling/building material and more recently as sleeping mats, bags, storage baskets, and trays [4]. Some edible species have been used in the human diet. For example, seeds of *Enhalus acoroides* (L.f.) Royle have been consumed by some asiatic ethnic groups (e.g., in Philippines and Indonesia) as a source of energy during fishing activity and for producing flour used in the preparation of biscuits [6]. Another known example of the human consumption of seagrasses is about *Zostera marina* L. Seeds of this species were used as a subsistence food by prehistoric inahbitants and Seri population of Mexico, and recently their employment in fine-dining restaurants has been shown [7]. A study also reported that the rhizomes of *Cymodocea* spp. have been included in the preparation of salad [8]. Given their high nutritional content and presence of compounds with phytochemical, nutraceutical, and bioactive properties, the interest in the use of seagrasses as functional food ingredients and in medicine has been recently increased [9,10,11,12,13]. Indeed, seagrasses contain many specialized metabolites, like polyphenols, terpenoids, and halogenated compounds, that are produced by plants as defense mechanisms against biotic (e.g., herbivory) and abiotic factors (e.g., low light, anoxic conditions) [14,15]. These molecules have interesting bioactive properties, such as antioxidant, anti-inflammatory, antimicrobial, antidiabetic, anti-obesity, antiproliferative, and anticancer activities, but information on their safety and efficacy is limited and requires further investigations [12,16]. *Cymodocea nodosa* (Ucria) Ascherson (common names: seahorse grass, little Neptune grass Sea) (Figure 1) is undoubtly among the most promising seagrass species as source of bioactive compounds and for the nutritional value [12,16]. This species belongs to the genus *Cymodocea* (family Cymodoceaceae), which includes three other dioecious species (*C. rotundata* Ascherson & Schweinfurth., *C. serrulata* (R.Brown) Ascherson & Magnus, *C. angustata* Ostenfeld) with a tropical-subtropical distribution [1]. These plants have herbaceous, monopodial, fleshy rhizomes with leaf bundles arranged on vertical rhizomes characterized by short internodes [17]. *Cymodocea nodosa* is the only species of the genus present in the Mediterranean Sea and its distribution spans over the whole basin and the coasts of the north-east Atlantic Ocean, from central Portugal to north-west Africa, including the Canary Islands. This fast-growing species can form both mono-specific and mixed meadows in shallow coastal habitats and transitional waters [4]. It is considered as a pioneer species due to its ability to quickly colonize bare areas of the seafloor favoring the establishment of *Posidonia oceanica* (L.) Delile, the endemic seagrass of the Mediterranean [4,18]. Up to date, no evidence of the use of *C. nodosa* for human consuption is available in literature according to our knowldege. The plant has recently been used as a model for evaluating the effects of a wide range of contaminants (e.g., trace elements, plastics, pharmaceuticals, heavy metals, and bisphenol A) present in marine environments [19,20,21,22,23,24] thanks to its ecological role, fast response times to environmental changes and capacity to grow under controlled conditions. *Cymodocea* nodosa can uptake heavy metals, such as Cd, Cu, Pb, and Zn, from the surronding environment (both water column and substrate) and accumulate them in different organs. For example, Zn is typically stored in leaves where it can reach concentration up to 60 µg/g DW, while in rhizome and roots concentrations are lower (up to 35 µg/g dried weight (DW)) [25,26]. However, this species can activate adaptive mechanisms to remove the excess of metals from their tissues [25].

Yet, the harvesting of large plant biomasses from natural populations to satisfy future market demand is not ecologically sustainable. Indeed, *C. nodosa* as other seagrass species is threatened by climate change related factors and anthropogenic stressors [27,28,29]. This species is currently included in the Annex 2 (List of Endangered or Threatened Species) of the SPA/BD Protocol of the Barcelona Convention, in the Annex I of the Bern Convention, and in the IUCN red list with the conservation status of “least concern” [30,31]. However, studies have shown that beached wrack consisting of detached leaves of *C. nodosa* could be a possible alternative source of biomass for producing bio-active compounds [32]. Moreover, this species can reproduce both by clonal propagation and seed. Studies demonstrated that large quantities of plants could be obtained in culture using in vitro propagation techniques and appositively designed aquaculture systems [22,33,34]. For example, by using a cultivation protocol under patent license in Italy, in which outdoor tanks are coupled with systems pumping continuously natural seawater, many *C. nodosa* plants can be produced in a sustainable manner by starting from relatively few seeds. Specifically, from two seeds collected from the natural environment two adult plants can be obtained, from which in turn at least 50 new plants each (approximately 1300 shoots with a rhizome network 28 m long) can be produced by cutting [34]. The high nutritional value and the presence of biologically active substances in its tissues referred to their dry weight make this species a potential source of compounds for dietary and pharmaceutical applications [12,16,32].

The present review aims to make a revision of existing literature on chemical constituents of *C. nodosa* and their biological activity.

## 2. Chemical Constituents of *Cymodocea nodosa*

Despite the growing interest in *C. nodosa* for playing a key ecological role and for being potential source of bioactive compounds, chemical studies about its composition are still limited. Interest in *C. nodosa* covers its potential nutritional and health-promoting properties due to its chemical constituents.

### 2.1. Mineral and Water Content

Several macro- and microelements are accumulated in *C. nodosa*, among which potassium (K) was found to be the most abundant (17,515 mg/g) followed by sodium (Na, 12,051 mg/g) in *C. nodosa* samples from the Montenegro Coast. Other elements were found in the following order: Mg > Ca > P > Si > Zn > Bi > Ba > Cr > Cu > Li. Interestingly, the low Na/K ratio (0.69) could be a positive factor in hypertensive subjects [35]. *C. nodosa* leaves collected from the coast of Chebba (Tunisia) contained remarkable levels mainly of Mg (35,101.7 µg/g DW), followed by K (12,330.5 µg/g DW), Ca (6805.5 µg/g DW), Na (4212.85 µg/g DW), and P (1529.65 µg/g DW). The trend in concentration of heavy metal was Zn > Cu > Ni > Pb, with Pb < 50 µg/g DW [10]. The water content of *C. nodosa* estimated as humidity was found to be 10 ± 0.59% using the gravimetric technique [36].

### 2.2. Carbohydrates

#### 2.2.1. Simple Sugars

Seagrasses are known to contain a considerable amount of soluble sugars, mainly sucrose [37]. *Cymodocea nodosa* leaves collected on the Straits of Messina (Sicily, Italy) and at Marsaxlokk (Malta) were found to contain a high content of sucrose and *myo*-inositol (more than 5% DW), together with *α*- and *β*-glucose, fructose, and other soluble carbohydrates [38]. Silva et al. [39] demonstrated that *C. nodosa* rhizomes from Ria Formosa coastal lagoon (South Portugal) contained about 3.5 fold more soluble sugars and four-fold more starch than seagrass *Zostera marina* L. while starch content in the leaves was identical in both plants. In addition, the sugar content in *C. nodosa* was not affected by light-limitation stress.

#### 2.2.2. Polysaccharides

*Cymodocea nodosa* samples from the southwestern coast of Montenegro were found to contain high level of *β*-glucans with a value of 13.04 ± 0.42 g/100 g, while α-glucans were 0.66 ± 0.17 g/100 g [35]. The first chemical investigation about sulfated polysaccharides extracted from *C. nodosa* was performed by Kolsi et al. [40,41] on plants collected on the coast of Chebba (Tunisia). A sulfated polysaccharide was isolated and characterized in its components, consisting in 23.17% of sulfate and 54.90% of total sugars. Among monosaccharides, galactose (44.89%), mannose (17.30%), arabinose (12.05%), xylose (9.18%), maltose (1.07%), together with uronic acid (11.03%) were identified.

### 2.3. Fatty Acids

Several studies reported *C. nodosa* as a good source of essential fatty acids important for human health and nutrition. The fatty acid composition of *C. nodosa* was investigated by [36] on samples collected from the Adriatic Sea, in southwestern Montenegro. *Cymodocea nodosa* was found to contain high levels of polyunsaturated fatty acids (PUFA) corresponding to 44.71% of total fatty acids (TFA), with *α*-linolenic and linoleic acids the most abundant (27.11% and 17.6%, respectively). These values were higher compared to those obtained from the seaweed *Halimeda tuna* (J. Ellis & Solander) J. V. Lamouroux, *Cystoseira barbata* (Stackhouse) C. Agardh and *Codium bursa* (Olivi) C. Agardh. High content of saturated fatty acid (SFA) was also found (38.34%) with high level of palmitic acid (33.11%), while monounsaturated fatty acids (MUFA) were 15.07% with oleic acid the main component (6.04%). These results were confirmed by Milović et al. in a further study [35]. A different trend was observed in *C. nodosa* leaves collected from the coast of Chebba (Tunisia), in which the most abundant fatty acid was found to be oleic acid (62.43%), followed by palmitic acid (21.65%) and linoleic acid (7.56%) [10].

*Cymodocea nodosa* leaves from northern and southern populations collected in Spain were found to be a rich source of fatty acids, with a total average value of 2.45 ± 0.3 mg/g DW of leaves, significantly higher compared to *P. oceanica* (1.80 ± 0.3 mg/g DW). PUFA represented 66.4 ± 1.1% of TFA with *α*-linolenic acid the most abundant, followed by linoleic acid. Monounsaturated fatty acids (MUFA) were 3.4 ± 0.5% of TFA being oleic acid the most represented, while saturated fatty acid (SFA) levels were 23.0 ± 1.0% of TFA with palmitic acid the main component. This study showed also a possible influence of global warming on the fatty acid production, with a reduction in PUFA and a raising in SFA levels [42].

### 2.4. Polyamines

Polyamines are metabolites occurring in plants, animals and microorganisms, characterized by the presence in their structure of two or more amino groups and occurring in free state or conjugated to small molecules or bounded to macromolecules. Their role in regulating physiological processes, as well as stress tolerance, is still under investigation [37,38]. Their distribution in seagrass C. nodosa was firstly investigated by Mariàn et al. [43] collecting the marine plant at Gando Bay (Gran Canaria, Canary Islands). Free and bound putrescine, spermidine, and spermine were quantified in roots, leaves, rhizomes, and apical zone, showing the meristematic tissue of the rhizome having a polyamine amount significantly higher compared to other organs (Table 1). The amount of polyamines was comparable to that found in terrestrial plants.

### 2.5. Sterols

Sterols produced by marine angiosperms are important components of diet of marine organisms. The first study investigating the sterol content of C. nodosa was performed in 1983 on plants collected at Port-Cros (France). The sterol fraction was obtained from a n-hexane extract of dry plants after saponification. Fractionation on silica gel column chromatography of the unsaponifiable fraction led to the purification of β-sitosterol (41.4% of total sterol) and stigmasterol (29.5%) as the most abundant, together with cholesterol (9.8%), campesterol (4.1%), Δ^5^-avenasterol (8.1%), and in minor amount trans-22-dehydrocholesterol (0.7%), brassicasterol (1.1%), desmosterol (0.7%), 24-methylene cholesterol (2.1%), and fucosterol (2.5%) [44]. These results were confirmed by a next study [45] in which ten phytosterols were obtained by fractionation of the unsaponifiable fraction from an acetone extract of fresh plant collected in the Bay of Naples (Italy). Cholesterol, campesterol, 22,23-dihydrobrassicasterol, brassicasterol, epibrassicasterol, sitosterol, stigmasterol, 5α-campestanol, 5α-ergostanol, and 5α-stigmastanol were purified and identified. Also in this case, sitosterol (40.8% relative abundance), stigmasterol (18.0%), and cholesterol (16.4%), were the most abundant components, similarly to terrestrial plants where these compounds are usually produced. The amount of β-sitosterol and β-stigmasterol in the unsaponifiable fraction of C. nodosa collected in Gulf of Boka Kotorska (Montenegro) was estimated as 5.70 ± 0.03 and 6.04 ± 0.08 µg/g, respectively [35]. Four 3-keto steroids with a 24-ethyl-cholestane side chain were further isolated from an apolar extract of C. nodosa collected at Agios Cosmas gulf near Athens (Greece) and identified as (20R)-22E-24-ethylcholesta-4,22-dien-3-one, (20R)-24-ethylcholest-4-en-3-one, (20R)-22E-6β-hydroxy-24-ethylcholesta-4,22-dien-3-one, and 6β-hydroxy-(20R)-24-ethylcholest-4-en-3-one [46]. These studies highlighted C. nodosa as rich source of phystosterols, valuable substances in marine food chain and bioactive agents possessing a wide range of pharmacological activity.

### 2.6. Phenols

The first evidence of the presence of phenolic compounds in *C. nodosa* was reported in 1979 for samples collected in the Bay of Naples (Italy), from which quercetin glucoside and isorhamnetin glucoside were isolated and identified [47]. A series of phenolics were detected and monitored by high performance liquid chromatography (HPLC) in *C. nodosa* samples near a volcanic CO_2_ vent on the Island of Vulcano (Italy): syringaldehyde, 4-hydroxybenzoic acid, gallic acid, vanillin, acetovinillone, coumaric acid, and ferulic acid were confirmed by commercial standards. Interestingly, the authors observed that the total content of phenolics decreased in concentration under high CO_2_/low pH conditions in this site [48].

Living and detrital specimen of *C. nodosa* from the Atlantic Ocean and Mediterranean Sea were analysed for their phenol content by HPLC coupled to photo diode array detector and mass spectrometry (HPLC-PDA-MS) many years later. Dried powdered leaves and rhizomes were extracted at room temperature with aqueous methanol (1:1) and the obtained extracts were compared in their chemical profiles. Among identified compounds, phenolic acids such as caftaric acid, chicoric acid (both confirmed by authentic samples), coutaric acid, and a dicoumaroyltartaric acid were found. Chicoric acid was the most abundant in all the extracts, showing values in a range of 8.13–27.44 mg/g DW in leaves and 0.31–3.70 mg/g DW both in rhizomes. Caftaric acid was present in smaller amount (0.20–2.92 mg/g DW) in leaves and in rhizomes (0.029–0.896 mg/g DW). Among flavonoids, quercetin and isorhamnetin glycosides were detected and identified by comparison with reference standards as quercetin 3-*O*-glucoside, quercetin 3-*O*-rutinoside (rutin), isorhamnetin 3-*O*-glucoside, and isorhamnetin 3-*O*-rutinoside [32]. These results were in part confirmed by a next study in which powdered samples of *C. nodosa* collected in Montenegro were extracted by cold maceration using a mixture of dichloromethane/methanol (1:1) as a solvent. The LC-UV-MS analyses showed the presence of coutaric acid, caftaric acid and diosmetin 7-sulfate. Diosmetin 7-sulfate was further isolated and confirmed by NMR data and reported in *C. nodosa* in this study for the first time. Furthermore, the total phenol content (TPC) was determined by Folin-Ciocalteu method on this extract showing a value of 37.82 ± 1.23 µg GA (gallic acid)/mg DW), a high content when compared to that obtained for other seaweeds species [36]. Based on HPLC-ESI-MS analysis of a hydrohalcoholic extract (EtOH-H_2_O 80% *v*/*v*) of *C. nodosa* leaves from Chebba coast (Tunisia), several phenolics were tentatively identified as catechin, quercetin 3-*O*-glucoside, quercetin 3-*O*-rutinoside, isorhamnetin 3-*O*-rutinoside, and derivatives of sinapic, ferulic and cinnamic acid not completely characterized [10,49]. In addition, in this study TPC was estimated to be 122.22 ± 13.36 µg GA/mg extract, while total flavonoid content (TFC) 81.50 ± 9.83 µg QE (quercetin equivalent)/mg extract) and condensed tannins 54.58 ± 13.65 µg CE (catechin equivalents)/mg extract, highlighting a high content in phenol constituents [10].

New findings emerged from a phytochemical study performed on *C. nodosa* rhizome collected in the Gulf of Pozzuoli (Naples). Except for isorhamnetin 3-*O*-glucoside previously reported, further constituents were isolated and characterized as isorhamnetin 3-*O*-galactoside, (−)-catechin, 4-(2,5-dihydroxyhexyl)benzene-1,2-diol, together with two new prenylated flavon-di-*O*-glycosides, 8-prenylnaringenin 5,7-*O*-*β*-diglucopyranoside and 5′-prenylchalco-naringenin 2′,4′-*O*-*β*-diglucopyranoside, named cymodiosides A and B, respectively. This study highlighted for the first time the presence of flavanones and prenylated flavonoids in seagrasses [50].

In a recent work, the chemical fingerprint of *n*-butanol extracts of *C. nodosa* whole plants was investigated by ultra-HPLC coupled to a high-resolution Orbitrap/MS giving a more comprehensive phenol profile. Plants were harvested in a site located in the Ligurian Sea (Italy) and grown in mesocosm containing natural seawater. A total of 39 compounds were tentatively identified, including citric acid, 23 phenolic acids and derivatives, 13 flavonoids, and two dihydrochalcones. This study confirmed the presence of previous reported metabolites but also revealed a larger chemical variety among the compound classes. Hydroxycinnamic acids were found free and in different conjugated forms not previously reported (*p*-coumaroyl hexoside, *p*-coumaroylmalic acid, *p*-coumaroylcaffeoyltartaric acid, feruloylhexoside, feruloyltartaric acid, diferuloyltartaric acid, feruloylmalic acid, caffeoylferuloyltartaric acid). The total amount of phenolic acids was estimated 357 ± 30 µg/g plant fresh weight (FW), being chicoric acid the most abundant (53.2 ± 3 µg/g FW). The total flavonoid content was 464 ± 22 µg/g FW with isorhamnetin hexoside the main component (226 ± 5 µg/g FW). Other isorhamnetin derivatives were detected in significant amount (rutinoside, acetylhexoside, and malonylhexoside), together with quercetin derivatives (rutinoside, hexoside, and malonylhexoside), kaempferol and naringenin hexosides. Two dihydrochalcones not previously reported, phlorizin and phloretin, enriched the phenol profile of *C. nodosa*. Finally, catechin, epicatechin and a procyanidin B-type dimer were tentatively identified. Interestingly, the metabolite production was influenced by a short-term exposure to ibuprofen as a response to oxidative stress induced by the drug [24]. An optimized extraction of phenols, by ultra sound assisted extraction for 30 min, from *C. nodosa* samples collected in Kheireddine region (Tunisia) led to obtain an EtOH-H_2_O (25%, *v*/*v*) extract containing TPC content of 113.07 µg GA equivalent/mg DW, TFC of 303.94 ± 1.45 µg QE/mg DW, and total condensed tannins of 172.85 ± 1.25 µg CE/mg DW. The chemical characterization of the extract by HPLC-PDA analysis revealed the presence of phenol constituents identified by comparison with reference standards. Among hydroxycinnamic acid, sinapic acid was the most abundant (0.741 ± 0.08 µg/mg DW) followed by ferulic acid and *trans*-cinnamic acid, while myricetin was the most represented flavonoid (0.620 ± 0.05 µg/mg DW) followed by quercetin 3-*O*-rutinoside (0.300 ± 0.05 µg/mg DW) [51].

**Figure 2:**
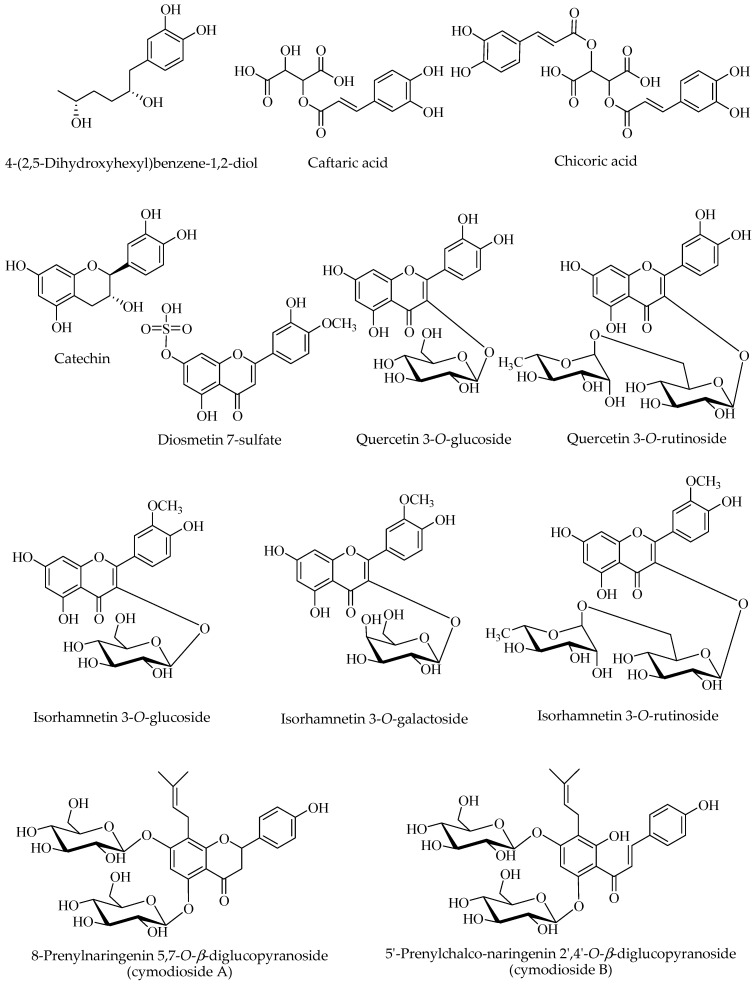
Major phenols isolated and/or identified in *Cymodocea nodosa* by NMR or by comparison with reference standards.

Chemical structures of fully identified phenols in *C. nodosa* are illustrated in Figure 2.

### 2.7. Terpenoids

**Figure 3:**
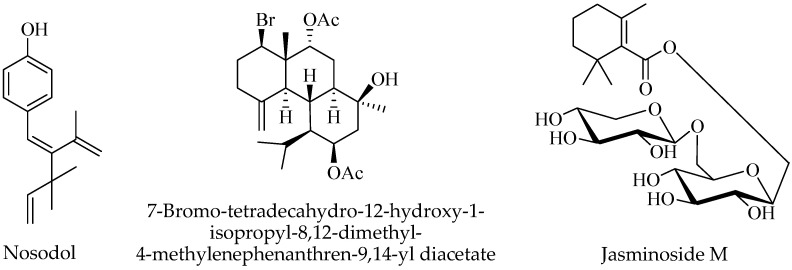
Terpenoids isolated from *Cymodocea nodosa*.

Existing chemical studies reported few terpenoid compounds occurring in *C. nodosa*. A new meroterpenoid named nodosol was isolated by Kontiza et al. [52], together with a new briarane diterpene named (1S*,2S*,3S*,7R*,8S*,9R*,11R*,12S*,14R*)-7-bromo-tetradecahydro-12-hydroxy-1-isopropyl-8,12-dimethyl-4-methylenephenanthren-9,14-yl diacetate (Figure 3). Both compounds were obtained from a dichloromethane–methanol (3:1) extract by samples collected at Porto Germeno in Corinthiakos Gulf (Greece) and identified by NMR, HR-MS, IR, and UV experiments. The monoterpenoid jasminoside M was isolated and identified by NMR data from *C. nodosa* rhizome collected in the Gulf of Pozzuoli (Naples) [50].

Carotenoids content was estimated in *C. nodosa* leaves collected from the coast of Chebba (Tunisia) resulting 0.929 ± 0.006 mg/g DW [10].

### 2.8. Diarylheptanoids

**Figure 4:**
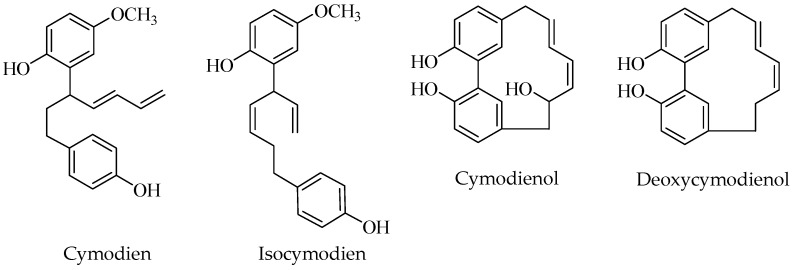
Diarylheptanoids isolated from *Cymodocea nodosa*.

Diarylheptanoids, a class on natural compounds characterized by a 1,7-diphenylheptane portion [53], were found as constituents of C. nodosa, as well as of marine organisms, by Kontiza et al. [54] for the first time. In this study, cymodienol (cyclic diarylheptanoid) and cymodiene (linear diarylheptanoid) were isolated from an apolar extract (dichloromethane–methanol, 3:1) and obtained as oils from C. nodosa samples collected in Agios Cosmas (Athens, Greece) (Figure 4).

Two other diarylheptanoids closely related to the previously described, deoxycymodienol and isocymodiene, were isolated in a further study by the same research group [52] from samples collected at Porto Germeno in Corinthiakos Gulf (Greece) and using the same extraction procedure (Figure 4). The structure of all compounds was established by 1D- and 2D-NMR, HR-MS, IR, and UV experiments. The occurrence of diarylheptanoids in marine organism was successively confirmed by the isolation of cymodienol in the seagrass *Z. marina* [55].

### 2.9. Volatilome

Biogenic volatile organic compounds (BVOCs) emission of *C. nodosa* samples collected in Carteau Cove (France) was evaluated by headspace solid-phase microextraction (HS-SPME) and analysed by gas chromatography-mass spectrometry (GC-MS). A total of 59 compounds were detected, consisting of alkanes (38.8%), esters (32.8%), sulfur compounds (11%), alkenes (6.9%), aldehydes (5.7%), ketones (3.2%), ethers (<1%), alcohols (<1%), and some unknown compounds (<1%). Fatty acid derivatives were estimated to be 81.5%, terpenoids 6.6%, and sulfur-containing compound 11.0%. Isopropyl myristate was the most represented compound (25.6%), emitted 10 to 37 times when compared to *P. oceanica*, *Zostera noltei* (Hornem.) Toml. & Posl., and *Z. marina*. Furthermore, *C. nodosa* was discriminated by some terpenoids such as camphor (0.24%), citral (1.23%), humulene (0.1%), and neral (0.86%). This study highligthed that seagrass can emit a wide variety of BVOCs, as observed in other marine organisms [56].

## 3. Therapeutic Perspectives

For thousands of years, seaweeds have been valued as an edible and health-enhancing resource, while seagrasses, deep-rooted marine angiosperms capable of completing their life cycle when submerged in water, are usually neglected especially for studies of potential bioactive compounds [57].

As previously reported, *C. nodosa* is a little studied plant as revealed by a very limited literature involving the nutritional and nutraceutical value of this species [6]. From a nutritional point of view, a sample harvest on the coast of Tunisia showed a relatively low quantity of lipids (about 5% (DW)), of which the most representative is oleic acid (about 62.0% of the total fatty acids); while the protein content was about 7.2% (DW). As concerns the high fiber content (56.4%), these are represented by sulfated polysaccharides, an attractive and heterogeneous class of candidate compounds for many therapies. Similar results have been found by other authors on *C. nodosa*; anyway, possible variations in macronutrients could be attributed to the seasonal periods and the different geographical area in which the marine plant grows. A significant quantity of pigment, and remarkable levels of minerals (among which calcium and magnesium) are detected, probably due to the bioaccumulation capacity of the marine plant [8,10]. The results suggest that this marine plant could be utilized as a healthy food item for human consumption; nevertheless the literature is still limited. Regarding the functional properties, *C. nodosa* demonstrated important swelling capacity values (6.71 ± 0.2 mL/g DW), the water retention capacity was equal to 12.26 ± 0.25 g water/g DW and oil retention capacity was 1.63 ± 0.03 g oil/g DW. These values are indicative of cohesiveness (the force which is necessary to attain a given deformation). Moreover, this marine plant presented a high firmness, springiness, and chewiness. The hydration properties of dietary fibres determine its optimal usage levels in foods because it is necessary to maintain a desirable texture [10].

### 3.1. Antioxidant Activity

Plants defend themselves against intracellular Reactive Oxygen Species (ROS) generation, synthetising specialized metabolites able to protect against ROS-induced cell damage and death. Among the classes of phytochemicals able to function as antioxidants, polyphenols are undoublty among the most important. Polyphenols are considered for their remarkable antioxidant properties; however, it is well-recognized their effects beyond this property. In fact, despite their unfavorable bioavailability, polyphenols have been shown to possess a plethora of intersting beneficial effects against processes involved in several age-related diseases, particularly diabete, neurodegenerative and cardiovascular ones [58]. In *C. nodosa* a high content of these compounds was described. In 2017, Kolsi et al. investigating the potential nutritional value associated to *C. nodosa* collected from Chebba (Tunisia), analysed a hydroethanolic extract characterized by a total phenolic concentration of 122.22 ± 13.36 mg GAE/g. The IC_50_ value of the extract, calculated with DPPH assay (0.33 mg/mL), was comparable to that of vitamin C used as control, and the IC_50_ determined by ABTS was in line with the ascorbic acid IC_50_ (40 mg/mL) [10,49].

An important antioxidant effect was also reported for polysaccharidic compounds from *C. nodosa*. Indeed, based on the molecular structure, degree, and length of branching and monosaccharide constituents, polysaccharides have been associated with antioxidant effects in numerous experimental conditions [59]. At concentration of 0.5 mg/mL their antioxidant activity measured by DPPH assay was very similar to that of vitamin C, with a scavenger activity of 82.44%. The same result was obtained using the ABTS test in which the plant polysaccharides showed 87.25% of antioxidant activity compared to 91.12% of vitamin C. Another technique that can be used to investigate the mechanism responsible for the antioxidant action is the quantification of the reducing power. The reducing power of *C. nodosa* polysaccharides was calculated performing the protocol published by Kumaran and Karunakaran that recorded the reduction of FeIII to FeII by monitoring the optical density variation induced by the antioxidant agent (OD) [60]. The *C. nodosa* polysaccharides tested at concentration of 1 mg/mL gave an OD = 0.3, in line with the data recorded for other polysaccharides reported in literature such as *Porphyra haitanensis*, *Ulva pertusa* and *Ulva lactuca* algae, reducing powers of 0.28 and 0.25 and 0.33 [61,62]. Usually, this effect is relate to the presence of polar groups such as -OH, thus the authors concluded that the *C. nodosa* polysaccharides structure was characterized by hydroxyl groups [63]. Moreover, Kolsi and colleagues observed an inhibition of the lipid peroxidation in a dose-dependent manner, with a significant reduction in the malonyldialdehyde (MDA) levels in HeLa cells subjected to oxidative stress induced by 100 mM Fe_2_SO_4_ [63]. Recently, Chaabani et al. developed a green extraction procedure and obtained a novel extract that resulted characterized by an high content of total polyphenols and flavonoids. Moreover, the eco-extract showed a great antioxidant power (1.9 μg/mL), strongly higher than the control compound Trolox (65 μg/mL) and it was not toxic on RAM 267.4 cells (up to 400 μg/mL). Thus, they studied the action of this extract in a cosmetic formulation. Interestingly, the addition of the lyophilized *C. nodosa* eco-extract to cream preparation increased the anti-aging action, strengthen the skin protection and revitalization of the cosmetic formulation. Moreover, the cream had a positive score at the sensor analysis and the formulation was stable during the 30 days of test [51].

### 3.2. Angiotensin Converting Enzyme (ACE) Inhibitory Activity

The inhibition of ACE is one of the most pursued therapeutic strategies for containing the blood pressure. In fact, the renin-angiotensin system plays a fundamental role in the regulation of blood pressure, electrolytes and blood volume, and in the pathophysiology of cardiovascular diseases [64]. Kolsi and collaborators demonstrated that an extract of *C. nodosa* has inhibitory effect in in vitro assays on the ACE enzymatic activity, with an IC_50_ value of 0.41 mg/mL, suggesting a possible role for a future application as a remedy for hypertension. A similar potency index was calculated using a sulfated polysaccharide extract from *C. nodosa*. Notably, the highest ACE inhibitory activity was observed at 0.8 mg/mL with an IC_50_ value of 0.43 mg/mL [40].

### 3.3. Antidiabetic Activity

Diabetes is one of the most common diseases in the world population, characterized by an increased glucose concentration in the blood (hyperglicemia). A therapeutic strategy for prevention and management of diabetes is decreasing blood glucose rise after a meal. Blunting carbohydrate intestinal digestion by inhibiting digestive enzymes such as *α*-amylase may be useful since the enzyme causes postprandial hyperglycaemia [65].

Kolsi’s research group, studying a hydroalcholic extract obtained from the whole powder plant of *C. nodosa*, demonstrated an appreciable concentration-dependent *α*-amylase inhibitory activity by this extract. The maximum inhibition was estimated to be 73.03% and the IC_50_ value was equal to 65.22 mg/mL. The modulation of the release rate of glucose from starch, although lower than that of the specific inhibitor acarbose (IC_50_ = 19.40 mg/mL), could contribute to the control of glycaemia; indeed it is generally recognized that carbohydrates are digested into oligosaccharides by *α*-amylase and then digested by gluco-amylase to maltose and maltotriose [49]. The authors supposed that a such activity can be associated with the presence of phenolic compounds, especially flavonoids. In this regard, several reports revealed that the activity of *α*-amylase is effectively inhibited by flavonoids probably for their ability to bind with proteins [66]. Moreover, by using an in vivo model of hyperglycaemia, obtained by an intraperitoneal treatment with alloxan 200 mg/kg for 2 weeks, the hydroalcholic extract of *C. nodosa* confirmed *α*-amylase inhibitory property and showed both significative hypoglycaemic effects and a protection of pancreas, kidney and liver dysfunctions, that typically are the main target of the diabetes-associated complications. Furthermore, *C. nodosa* extract was able to restore the lipidic profile, changed after alloxan-treatment, decreasing the triglyceride, low density lipoprotein cholesterol (LDL-C) and total cholesterol rates in the plasma of diabetic rats by 46%, 35%, and 21%, respectively, and increased the high density lipoprotein cholesterol (HDL-C) level by 36%, which helped to maintain the homeostasis of blood lipids [66]. Very similar results were obtained 2 years before by the same research group testing on alloxan-induced diabetic rats a crude sulphated polysaccharide extract of *C. nodosa* [41].

Another interesting bioactive compound in *C. nodosa* is chicoric acid, a phenolic compound of special interest owing to its large spectrum of biological properties [32]. *Cymodocea nodosa* has been described as a promising and renewable source of chicoric acid that is a dicaffeyltartaric acid endowed with potential nutraceutical value in the management of metabolic alterations; in particular, several recent papers highlight its role in the regulation of hyperglycaemia and dyslipidaemia through the activation of the AMP-activated protein Kinase (AMPK) pathway [67,68,69,70,71]. This pathway has a recognised role in the control of the energetic metabolism of cells, representing a target for the prevention and treatment of diabetes and its complications [72].

### 3.4. Regulation of Lipidic Profile

As already mentioned in the previous paragraph, the hydroalcholic extract of *C. nodosa* in a diabetic animal model is able to improve the lipidic profile. Another interesting paper from Kolsi and collaborators [73] showed that the crude sulfated polysaccharides isolated from the roots of *C. nodosa* and orally administered to rats fed with a high cholesterol-high fat diet (HFD) for 6 weeks induced a decrease in body weight gain compared to the cholesterol-fed-control; it also regulated the lipidic profile, as observed by the significant decrease in serum levels of cholesterol, triacylglycerols and LDL-C levels by 45%, 53% and 25% respectively, and by the increase in HDL-C level by 19%, protecting hepatic and renal function, too. Worthy of mentioning, the authors, speculating on the possible mechanisms underlaying these beneficial effects, observed that the sulfated polysaccharides were endowed with inhibitory activity on lipase enzyme. Either in the serum and intestine of HFD-animals supplemented with the polysaccharide extract, the lipase activity was markedly reduced by 40% and 45% respectively, compared to HFD-animals resulting almost superimposable with the control ones. Such a result is indicative of reduced fat absorption at intestinal level; in agreement with its physiological role, the pancreatic lipase is a key enzyme for the hydrolysis of triglyceride into absorbable monoglycerides and free fatty acids.

### 3.5. Antibacterial and Antifungal Activities

Anti-microbial activity of plant polysaccharides are well recognised [74,75]. The sulfated polysaccharidic extract obtained from *C. nodosa* roots by Kolsi et al. [73] was also studied in vitro to investigate its antibacterial and antifungal profile. The polysaccharide showed a good activity against Gram^+^ bacteria *Staphylococcus aureus* and *Micrococcus luteus*, with a MIC values (6.25 to 50 mg/mL) comparable to those found for other marine polysaccharides [76,77]. Nodosol, isolated by Kontiza’s group, displayed a potent inhibitory activity with a MIC value of 16 mg/mL against *Mycobacterium fortuitum*, *Mycobacterium phlei*, and *Mycobacterium smegmatis* [52].

Regarding the polysaccharide antifungal activity, the main effect was revealed against *Candida albicans* (MIC = 12.5 mg/mL, followed by *Aspergillus niger* (MIC = 6.25 mg/mL) and *Fusarium oxysporum* (MIC = 12.5 mg/mL), while no activity was observed against *Saccharomyces cerevisiae* [63].

### 3.6. Antiproliferative Activity

A potential anticancer activity of the *C. nodosa* polysaccharidic extract by Kolsi et al. was also reported. In particular, the antiproliferative effect was revealed on human cancer cultures (Hela cell from a cervical tumor). The MTT test, performed at 72 h, showed that the polysaccharides extract exhibited a significant and dose-dependent cytotoxic effect and at 0.5 mg/mL the highest percentage of cytotoxic effect (58.33 ± 2.13) was found [63].

Milović and collaborators investigated several seaweeds extracts, harvested in the Adriatic coast, exploring potential antitumor activity and interestingly *C. nodosa* extract demonstrated strong cytotoxicity against Hela cells (IC_50_ = 13.28 ± 0.39 μg/mL) and human chronic myelogenous leukaemia cell line, K562 (IC_50_ = 19.64 ± 1.55 μg/mL) [36]. Two keto-steroids and a diarylheptanoid derivative (cymodienol), isolated from *C. nodosa*, exhibited significant cytotoxicity against NSCLC-N6 lung cancer cell lines [46,54].

## 4. Conclusions

The marine habitat represents a great opportunity for research of new organisms and new molecules for pharmaceutical and nutraceutical applications. Inside Mediterranean basin a great biodiversity is present which *C. nodosa* belongs to. Indeed, this seagrass is among the most spread plant species in the Mediterraneum sea. Moreover, particularly interesting is the possibility to produce this plant in a way that by-passes the problem of sea supply, i.e., by aquaculture systems, opening the opportunity of large scale cultivation as already happens for algae.

Several papers have reported in *C. nodosa* the presence of high value molecules for health whose extraction in some cases has been successfully performed with eco-sustainable techniques. Most of the studies carried out on *C. nodosa* chemical composition has been performed in the last twenty years, firstly focused on plant response at environmental stress and changes. *Cymodocea nodosa* emerged as a rich source of polyunsaturated fatty acids and phytosterols, as well as phenols, including phenolic acids, hydroxycinnamic acids with chicoric and caftaric acids the most represented, and flavonoid glycosides, mainly quercetin, isorhamnetin, and naringenin derivatives. Four diarylheptanoids were found as *C. nodosa* constituents for the first time, while terpenoids are less represented, with only two new isolated compounds. Few compounds were isolated and characterized by both NMR and MS analyses, while a larger number of metabolites was detected by high-sensitive analytic techniques such as HPLC-PDA or HPLC-MS and confirmed in some cases by injection of reference standards. Overall, the existing studies provide a good overview of *C. nodosa* specialized metabolism, with most of the constituents shared with terrestrial plants. Further studies are advisable to provide a more comprehensive chemical profile of this emerging source of bioactive agents.

Currently, only a limited number of investigations have reported pharmacological effects about extracts or isolated molecules from *C. nodosa*. Nevertheless, some interesting evidence already suggest this seagrass as an organism which may be used as biomass or as source of compounds for the prevention/treatment of diseases. In particular, the plant demonstrated antioxidant, antihypertensive, cytotoxic activity against cancer cells or infectious microorganisms and protection against metabolic disorders. As critical points, studies were mostly focused on the polyphenolic and the polysaccharidic extracts. Though based on this evidence it could be suppose that these constituents are mainly associated to the healthy profile of this seagrass; nevertheless an accurate characterization of the phytochemical composition is almost always missing; therefore it is not currently possible to establish the main constituent/s responsible for the beneficial effects. Moreover, no clinical studies were performed. Moreover, even if its cultivation may allow to exclude safety concerns linked to environmental contaminants, further studies investigating the toxicity profile of the plant are to be strongly considered looking at a safe nutraceutical use.

In conclusion, although this review shows a nutraceutical potential of *C. nodosa*, highlights also the need for further studies about its chemical composition and pharmaco/toxicological activities to give more insights on the relevance of this seagrass for health support and the best dosage to be administered under a standardized chemical profile.

## Figures and Tables

**Table 1:** Polyamine levels (nmol/g) in the apical zone of *Cymodocea nodosa*.

	Putrescine	Spermidine	Spermine
Free	1583 ± 147	19 ± 1.4	0.8 ± 0.1
Bound-soluble	25,886 ± 488	35 ± 2.05	63 ± 2.99
Bound-unsoluble	7530 ± 136	3.29 ± 0.14	8.43 ± 0.39
